# A multi-target protective effect of Danggui-Shaoyao-San on the vascular endothelium of atherosclerotic mice

**DOI:** 10.1186/s12906-023-03883-3

**Published:** 2023-02-20

**Authors:** Yuemeng Sun, Yushan Gao, Lu Zhou, Yixing Lu, Yulin Zong, Haoyu Zhu, Yang Tang, Fengjie Zheng, Yan Sun, Yuhang Li

**Affiliations:** School of Chinese Medicine, Beijing University of Chinese Medicine, Beijing, 100029 China

**Keywords:** Danggui-Shaoyao-San, Atherosclerosis, Vascular endothelium, Multiple targets, Interα5β1/c-Abl/YAP

## Abstract

**Background:**

Atherosclerosis (AS) is a chronic disease characterized by abnormal blood lipid metabolism, inflammation and vascular endothelial injury. Vascular endothelial injury is the initial stage during the occurrence of AS. However, the function and mechanism of anti-AS are not well characterized. Danggui-Shaoyao-San (DGSY) is a classic Traditional Chinese Medicine (TCM) prescription for the treatment of gynecological diseases, and has been widely used in the treatment of AS in recent years.

**Methods:**

ApoE^−/−^ atherosclerosis male mice were established by feeding with high-fat diet, and then randomly divided into three groups: Atherosclerosis group (AS), Danggui-Shaoyao-San group (DGSY), and Atorvastatin calcium group (X). The mice were administered with the drugs for 16 weeks. Pathological changes in aortic vessels were examined by staining with Oil red O, Masson and hematoxylin–eosin. In addition, blood lipids were analyzed. The level of IL-6 and IL-8 in aortic vessels were detected by ELISA and the expression of ICAM-1 and VCAM-1 in the aortic vascular endothelium were measured by Immunohistochemical. The mRNA expression of interα5β1/c-Abl/YAP in the aortic vessels were measured by Real-time quantitative PCR and location of expression was assessed by immunofluorescence.

**Results:**

DGSY can significantly reduce the content of TC,TG and LDL-C and increase the level of HDL-C in the serum, reduce the plaque area and inhibit the concentration of IL-6 and IL-8, down-regulate the expression of IVAM-1,VCAM-1 and interα5β1/ c-Abl/YAP in the aortic vessels.

**Conclusions:**

Collectively, DGSY can alleviate vascular endothelium damage and delay the occurrence of AS, and the underlying mechanism may be related to the multi-target protective of DGSY.

## Introduction

Atherosclerosis (AS), as a pathological basis of cardiovascular and cerebrovascular diseases, is the main cause of ischemic heart disease and stroke [1]. It is characterized by abnormal lipid metabolism, inflammatory reactions, vascular endothelial dysfunction, and plaque formation [2]. Globally, almost 20 million people die from AS-related diseases every year making it a serious threat to human health. In Europe, four million people die from cardiovascular diseases every year [3]. Moreover, AS reduces the quality of life of patients, creating challenges for their immediate society [4, 5]. Therefore, it is imperative to either prevent or delay AS progression. Although the pathogenesis of AS is not well understood, vascular endothelial dysfunction has been linked to the occurrence of AS [6]. Endothelial barrier dysfunction leads to lipid deposition in the vascular wall and the invasion of inflammatory cells [7], resulting in plaque formation. Therefore, protection of the vascular endothelium is an effective method to prevent or treat AS.

Danggui-Shaoyao-San (DGSY) is a classic prescription of traditional Chinese medicine. DGSY is composed of six medicinal herbs — Danggui (*Angelica Sinensis Ridix*), Baishao (*Paeoniae Radix Alba)*, Fuling (*Poria*), Chuanxiong (*Chuanxiong Rhizoma*), Zexie (*Alismatis Rhizoma*) and Baizhu (*Atractylodis Macrocephalae Rhizoma*) — in the ratio 9:48:12:24:24:12. DGSY has recently been used in the treatment of AS [8–11]. Moreover, DGSY reduced total cholesterol (TC), triglyceride (TG), and low-density lipoprotein (LDL), and increased high-density lipoprotein (HDL) in gestational hypertension murine models [12]. Similarly, DGSY reduced blood viscosity and red blood cell aggregation in rabbits; improved hemorheology abnormalities; reduced the expression of AngII and AT1R in podocytes from rats with nephrotic syndrome; improved proteinuria [13, 14] as well as downregulated the specific expression of vascular adhesion molecule 1 (VCAM-1) mRNA in dyslipidemia; exhibited anti-monocyte adhesion; and protected vascular endothelial function [15]. Furthermore, ferulic, paeoniflorin, and poria acids, the active ingredients in DGSY, inhibited the secretion of IL-6 and TNF-α, and reduced cellular inflammation [16]. Although numerous experimental studies have provided a pharmacological basis for the treatment of AS with DGSY, its detailed mechanism of action is yet to be fully elucidated. Despite the various drugs for the treatment of AS, their side effects limit their clinical application [17, 18]. Conversely, traditional Chinese herbal medicines like DGSY are characterized by few side effects and low toxicity [19–21], probably resulting in the recent interest in their use for the treatment of AS.

In the present study, we determined the multi-target protective effect of DGSY on vascular endothelium in an AS murine model. Specifically, we determined its regulatory effect on the interα5β1/c-Abl/YAP turbulence signal associated with vascular endothelial dysfunction to reveal potential mechanisms of AS. This study establishes the clinical application of DGSY for the treatment of vascular endothelial damage and the prevention of AS.

## Materials and methods

### Animal rearing and treatment

Mice were reared independently in ventilation cage systems of the animal room of the Beijing University of Chinese Medicine at 25(± 2) ℃ and relative humidity of 40%-60% under a 12-h day and 12-h night cycle. Both food and water were freely availed to the mice. All experimental protocols were approved by the Animal Ethics Committee of Beijing University of Chinese Medicine (Approval Batch No: BUCM-4–2021060802-2056). After adaptive feeding for a week, ApoE-/- mice were randomly divided into three groups (n 1/3 12 each): Model group (M), Danggui-Shaoyao-San group (DGSY), and Atorvastatin calcium group (X). All were fed a high-fat diet (15% lard, 2% cholesterol, and 0.05% cholic acid) from Beijing Vital River Laboratory Animal Technology. Twelve C57BL/6 J mice served as the normal control group (NC) and were fed a conventional diet throughout the experimental period. Mice were continuously fed for 16 weeks to establish the AS model. In the first week of the experiment, the DGSY group was administered a 16.77 g/kg/d dose of DGSY intragastrically, and the X group was given a 5 mg/kg/d dose of atorvastatin calcium tablets (H20051408, New York, United States) intragastrically. The NC and the AS groups were given a similar dose of distilled water intragastrically. After treatment for 16 weeks, the mice were sacrificed via overdoses of sodium pentobarbital. Blood samples were obtained, six aortic vessels were dissected, fixed with 4% paraformaldehyde, and stored at 4℃ (BCD-315TNGS, Qingdao, China) for subsequent experimentations. Moreover, six aortic vessels from each group were stored at -80℃ (ULTS1490, Suzhou, China).

### Preparation of drugs

Atorvastatin calcium tablets were ground into powder and dissolved in distilled water when required.

**Fig. 1:**
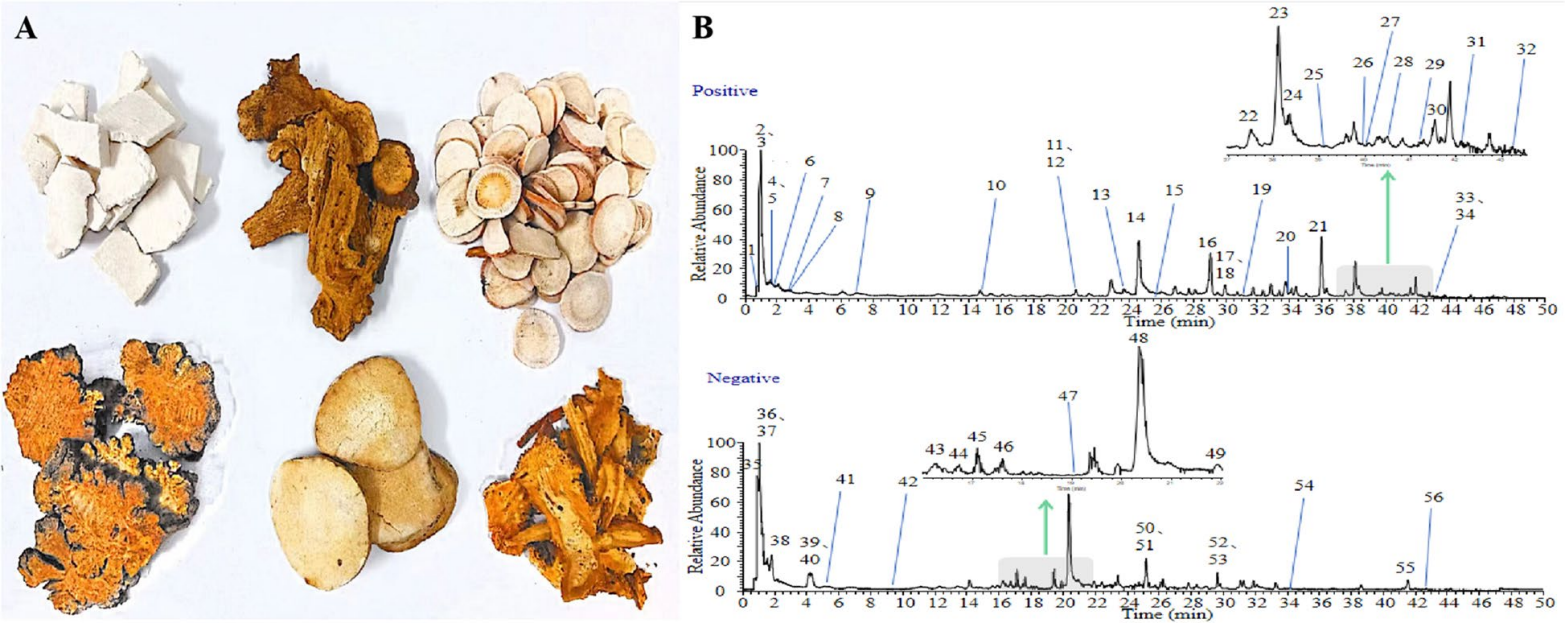
**A** Herbal composition of DGSY. **B** Total ion chromatogram of DGSY

DGSY was purchased from Beijing Tong Ren Tang Jingxi Pharmacy Co., LTD (Beijing, China) and is shown in Fig. 1. The crude herbs constituted with six herbs[10]and their proportions were shown in Table 1.

**Table 1:** Compositions of medicinal herbs in DSGY

Name of medicine herbs	DGSY(g)
*Angelica Sinensis Ridix*	9
*Paeoniae Radix Alba*	48
*Poria*	12
*Chuanxiong Rhizoma*	24
*Alismatis Rhizoma*	24
*Atractylodis Macrocephalae Rhizoma*	12

These herbs were first placed in an AI electric earthenware pot (GX-500A, Guangdong, China) and soaked for 30 min in distilled water, before boiling at medium heat for 30 min to obtain the decoction which was then decanted. Subsequently, 1000 ml water was added to the herbs and the process was repeated. The two drug decoctions were mixed and concentrated to 2 g/ml and then stored at 4℃.

### Ultra-high performance liquid chromatography-mass spectrometry analysis (UHPLC-MS)

The DGSY solution was extracted in methanol, and the supernatant was filtered through 0.22 μm microporous membranes. This was injected into a UHPLC-LTQ-Orbitrap-MS system.

### Oil red O Staining

Frozen aortic vessels were cut into 10 µm slices (CM 1950, Leica, Germany), air-dried, then stained with Oil Red O (G1261, Solarbio, China) according to the manufacturer’s instructions. To evaluate the effect of drug administration, the ORO-stained slides were observed and recorded under a positive fluorescence microscope (DM4B, Leica, Germany) (100x), then marked and assessed with Image-Pro Plus version 6.0 analysis software (Media Cybernetics, Maryland, United States).

### Masson’s trichrome staining

Frozen aortic vessel slides were air-dried and then stained with Masson’s trichrome dye (G1345, Solarbio, China) according to the manufacturer’s instructions. The vascular morphological changes were observed and recorded under a positive fluorescence microscope (100x, 200x).

### Hematoxylin and eosin staining

Frozen aortic vessel slides were obtained from each group and dried on a bench and then stained with hematoxylin and eosin (HE, KIT-9706, MXB, Fujian, China). Morphological changes in aortic vessels were observed and recorded under a positive fluorescence microscope (100x, 200x).

### Measurements for serum biochemical markers

Retro-orbital bleeding was collected into 1.5 ml centrifuge tubes and stored at 4℃ for 4 h. Samples were then centrifuged (3000 rpm, 10 min) and the supernatants were collected. In these, the levels of TC, TG, LDL-C, and HDL-C were measured by a vertical automatic blood biochemical analyzer (7080, HITACHI, Shanghai, China).

### ELISA analysis

Aortic vessel tissue samples were ground with a high throughput cryogenic tissue grinder (Scientz-48L, Ningbo, China) to obtain homogenates which were then centrifuged at 12000 rpm for 15 min at 4℃ (Eppendorf 5425 R, Hamburg, Germany). IL-6 (1203210413, RayBio, Atlanta, United States) and IL-8 (M211224-104a, Neobioscience, Shenzhen, China) were assayed according to the manufacturer’s instructions, and the absorbance was measured at 450 nm.

### Immunohistochemical analysis

Frozen aortic vessel slides were incubated at 95 °C for 10 min in sodium citrate for thermal antigen repair, before rinsing thrice with PBS —each time for 5 min— and incubating with goat serum for 10 min. Slides were then incubated with the primary antibody (intercellular adhesion molecule 1 (ICAM-1) (1:280); VCAM-1 (1:50) at 4℃ overnight. Then the slides were incubated with a secondary antibody for 1 h and visualized with DAB (2109290031H, MXB, Fujian, China). The sections were observed under a positive fluorescence microscope to assess the loci of positive expression and the depth of staining. Positive expression was denoted by either yellow or brownish-yellow stained particles. The sections were recorded with Image-Pro Plus version 6.0 analysis software.

### Real-time quantitative PCR analysis

Total RNA was extracted from aortic vessels using Trizol (15,596,018, Invitrogen, California, United States) using the reverse transcription kit (K1622, Thermo, Massachusetts, United States) in line with the manufacturer’s instructions. The concentration and purity were measured and samples were reverse-transcribed to cDNA. The PCR primers targeting *interα5β*, *c-Ab1*, *YAP,* and *GAPDH* genes are shown in Table 2. The PCR cycling conditions were: one cycle at 95 °C for 5 min, then 40 cycles for 95 °C for 15 s, 60 °C for 20 s, and 72 °C for 30 s. The relative expression of target genes was calculated by 2^−ΔΔCT^.

**Table 2:** Primer sequences used for qPCR

genes	Forward Primer (5'-3')	Reverse Primer (5'-3')
*Inter α5β1*	GGCTATGTCACCGTCCTTAAT	CTAGCCCATCTCCATTGGTATC
*YAP*	GAAAGGGCTCTAGTGGGTAAAG	AAATCAGGCTAAGGGAAGTAAGG
*c-Ab1*	GGAGTATTGCTCTGGGAGATTG	GCTCCATGCGGTAGTCTTT
*GAPDH*	AACAGCAACTCCCACTCTTC	CCTGTTGCTGTAGCCGTATT

### Immunofluorescence staining

Frozen aortic vessel slides were incubated in sodium citrate at 95 °C for 10 min for thermal antigen repair. interα5β1(1:100), c-Abl (1:50), and YAP (1:50) were used as primary antibodies and goat anti-rabbit IgG was used as the secondary antibody. After reactions with the primary antibodies, samples were incubated overnight at 4℃ before the fluorescent secondary antibody was added. Both samples were diluted with PBS (1:200) followed by incubation for 1 h. Then, a drop of anti-fluorescence quencher containing DAPI (GR3405163-3, Abcam, Cambridge, United Kingdom) was added to cover the glass seal. The sections were observed under a positive fluorescence microscope.

### Data analysis

SPSS software v.22 was used for statistical analysis. All data were expressed as mean ± standard deviation (SD). One-way ANOVA was used to compare multiple groups. The LSD method was used to compare the differences between the groups when the variance was homogeneous. The Games-Howell method was used to compare differences between groups when the variance was not uniform. A value of *p* < 0.05 indicated that the difference was statistically significant.

## Results

### DGSY Components

DGSY samples were analyzed after chromatographic separation due to the optimized LC–ESI–MS method. There were 56 peaks in chromatograms of DGSY samples, which consisted of ferulic acid, ligustrolide, paeoniflorin, and other compounds as shown in Fig. 1 and Table 3.

**Table 3:** The main chemical components of DGSY

PM	tR/min	Molecular formula	EV	Error(ppm)	Secondary fragment(MS/MS)	compound name
1	0.88	C_6_H_14_N_4_O_2_	174.111970	1.70	158.0、130.10、116.07、70.07、60.06	Arginine
2	0.97	C_5_H_9_NO_4_	147.053410	1.70	130.05、102.6、84.05、68.75	Glutamic acid
3	0.98	C_12_H_22_O_11_	342.116530	0.94	233.5、191.22、145.0、127.04、85.03	sucrose
4	1.53	C_9_H_13_N_3_O_5_	243.086080	2.29	226.12、198.11、141.05、112.05、70.07	Cytidine
5	1.54	C_5_H_5_N_5_	135.054920	3.13	119.04、94.04、70.62	Adenine
6	1.66	C_6_H_5_NO_2_	123.032570	4.42	124.04、96.05、80.05	Nicotinic acid
7	2.62	C_9_H_11_NO_3_	181.074420	2.90	165.06、147.04、136.08、123.04、119.05	L-Tyrosine
8	2.66	C_6_H_13_NO_2_	131.095110	3.67	118.94、87.10、86.10、69.07	Leucine
9	6.94	C_9_H_11_NO_2_	165.079530	3.34	149.06、131.05、120.08、103.05	Phenylalanine
10	14.70	C_11_H_12_N_2_O_2_	204.090530	3.21	188.07、159.09、146.06、118.07	Tryptophan
11	20.66	C_9_H_6_O_3_	162.032170	2.92	145.03、135.04、117.03、107.03、89.04	7-Hydroxycoumarine
12	20.66	C_16_H_18_O_9_	354.095960	2.49	229.91、163.04、145.03、117.03	Chlorogenic acid
13	23.85	C_10_H_11_NO	161.084570	3.12	144.08、134.10、120.08	3-(2-Hydroxyethyl)indole
14	24.57	C_10_H_12_O_2_	164.084180	2.77	141.06、137.10、105.07、79.06	4-Phenylbutyric acid
15	25.47	C_10_H_8_O_4_	192.042990	3.79	178.03、165.06、149.06、137.06、133.03	Scopoletin
16	29.08	C_12_H_16_O_4_	224.105390	2.39	207.10、189.09、161.10、119.09、105.07、91.05	Senkyunolide H or Senkyunolide I
17	30.38	C_11_H_14_O_2_	178.099980	3.36	161.10、143.09、133.10、119.09、105.07、91.06	Butyl benzoate
18	30.47	C_12_H_18_O_4_	226.121113	3.07	209.12、191.11、153.06	Senkyunolide J
19	31.04	C_11_H_6_O_3_	186.032270	3.12	169.07、143.05、131.05、115.05	Psoralen
20	33.99	C_15_H_24_O	220.183280	2.54	221.19、203.18、163.15、147.12、133.10、107.09	alismol
21	35.98	C_12_H_16_O_2_	192.115570	2.82	175.11、147.12、137.06、91.05	Senkyunolide A
22	37.47	C_15_H_20_O	216.152113	3.23	199.14、189.16、175.15、145.10、131.08	Atractylon
23	38.11	C_12_H_14_O_2_	190.099810	2.26	191.11、173.10、163.11、145.10、117.07、91.05	Ligustilide
24	38.35	C_12_H_18_O_2_	194.131270	3.06	177.13、149.13、125.06、107.09、97.07、79.06	Sedanolide
25	39.13	C_15_H_26_O_2_	238.193860	2.45	203.18、163.15、147.12、107.09	Alismoxide
26	39.77	C_15_H_20_O_2_	232.146990	2.84	233.15、215.14、187.15、177.09、151.08	Atractylenolide II
27	40.09	C_30_H_46_O_5_	486.336170	3.38	451.32、397.27、353.24、201.13	Alisol C
28	40.50	C_32_H_48_O_6_	528.346783	3.22	469.33、451.32、433.31、381.27、353.25	23-O-Acetylalisol C
29	41.29	C_15_H_18_O_2_	230.131363	2.97	231.13864、185、13	Atractylenolide I
30	41.68	C_18_H_30_O_2_	278.225240	2.36	274.27、226.95、219.18、191.11	Linolenic acid
31	42.11	C_16_H_22_O_4_	278.152450	2.31	149.02、121.10、109.10、95.09、81.07、67.06	Dibutyl phthalate
32	43.25	C_32_H_50_O_5_	514.367300	2.86	478.75、352.08、274.88、201.16、159.12、121.10	23-O-Acetylalisol B
33	43.66	C_30_H_48_O_4_	472.356520	2.68	415.32、348.38、308.74、201.16	Alisol B
34	43.67	C_32_H_48_O_5_	512.349413	-1.49	513.36、449.04、425.97、337.85	Alisol O
35	0.91	C_4_H_7_NO_4_	133.037750	1.81	115.00、95.03、88.04、71.01	Aspartic acid
36	0.94	C_6_H_14_O_6_	182.079360	1.79	163.06、119.04、101.02、96.97、89.02、71.01	Mannitol
37	0.98	C_6_H_12_O_6_	180.063670	1.58	161.05、131.04、89.02、85.03、71.01、59.01	Fructose
38	1.78	C_6_H_8_O_7_	192.027340	1.77	173.26、129.02、111.01、87.01、85.03	Citric acid
39	4.12	C_6_H_6_O_3_	126.031880	1.49	107.29、107.29、100.03、97.03	Pyrogallol
40	4.18	C_7_H_6_O_5_	170.021517	-0.04	169.01、125.02	Gallic acid
41	4.45	C_10_H_13_N_5_O_4_	267.097510	2.83	266.09、134.05、119.04	Adenosine
42	9.63	C_7_H_6_O_4_	154.026917	2.00	153.02、109.03	protocatechuic acid
43	16.24	C_8_H_8_O_4_	168.042477	1.30	123.05、121.03、101.37、95.05、68.80	Vanillin
44	16.74	C_15_H_14_O_6_	290.079790	2.59	271.06、203.07、151.04、125.02、109.03	Catechin
45	17.18	C_23_H_28_O_12_	496.158067	-0.02	407.66、281.07、165.06、137.02	oxypaeoniflora
46	17.61	C_16_H_18_O_9_	354.095870	2.22	280.83、191.06、179.04、173.05、135.05、93.03	Neochlorogenic acid
47	19.18	C_8_H_8_O_3_	152.047640	1.92	136.02、123.05、108.02、95.01	Vanillin
48	20.37	C_23_H_28_O_11_	480.164090	1.93	466.12、358.34、327.11、165.06、121.03	albiflorin
49	21.93	C_10_H_10_O_4_	194.058200	1.48	178.03、149.06、137.02、134.04	ferulic acid
50	25.04	C_9_H_16_O_4_	188.105190	1.77	187.10、169.09、143.11、125.10、97.07	Azelaic acid
51	25.19	C_23_H_28_O_11_	480.164320	1.27	433.24、371.50、327.86、221.02、151.08、121.03	Paeoniflorin
52	28.98	C_20_H_20_O_6_	356.126877	2.49	340.10、296.11、281.08、219.07、175.08、147.08	coniferyl ferulate
53	29.30	C_15_H_12_O_5_	272.069170	2.55	271.06、203.07、159.08、130.04	Naringenin
54	34.03	C_15_H_10_O_4_	254.058520	2.39	263.05、209.06、107.01	Chrysin
55	41.54	C_31_H_48_O_4_	484.356427	2.41	483.35、423.33、409.28、310.00、128.12	Dehydrotumulosic acid
56	42.68	C_31_H_46_O_4_	482.340747	2.36	467.71、421.31、403.30、311.20、271.17、97.97	Polyporenic acid C

### Effects of DGSY on pathological morphology of aortic vessels

**Fig. 2:**
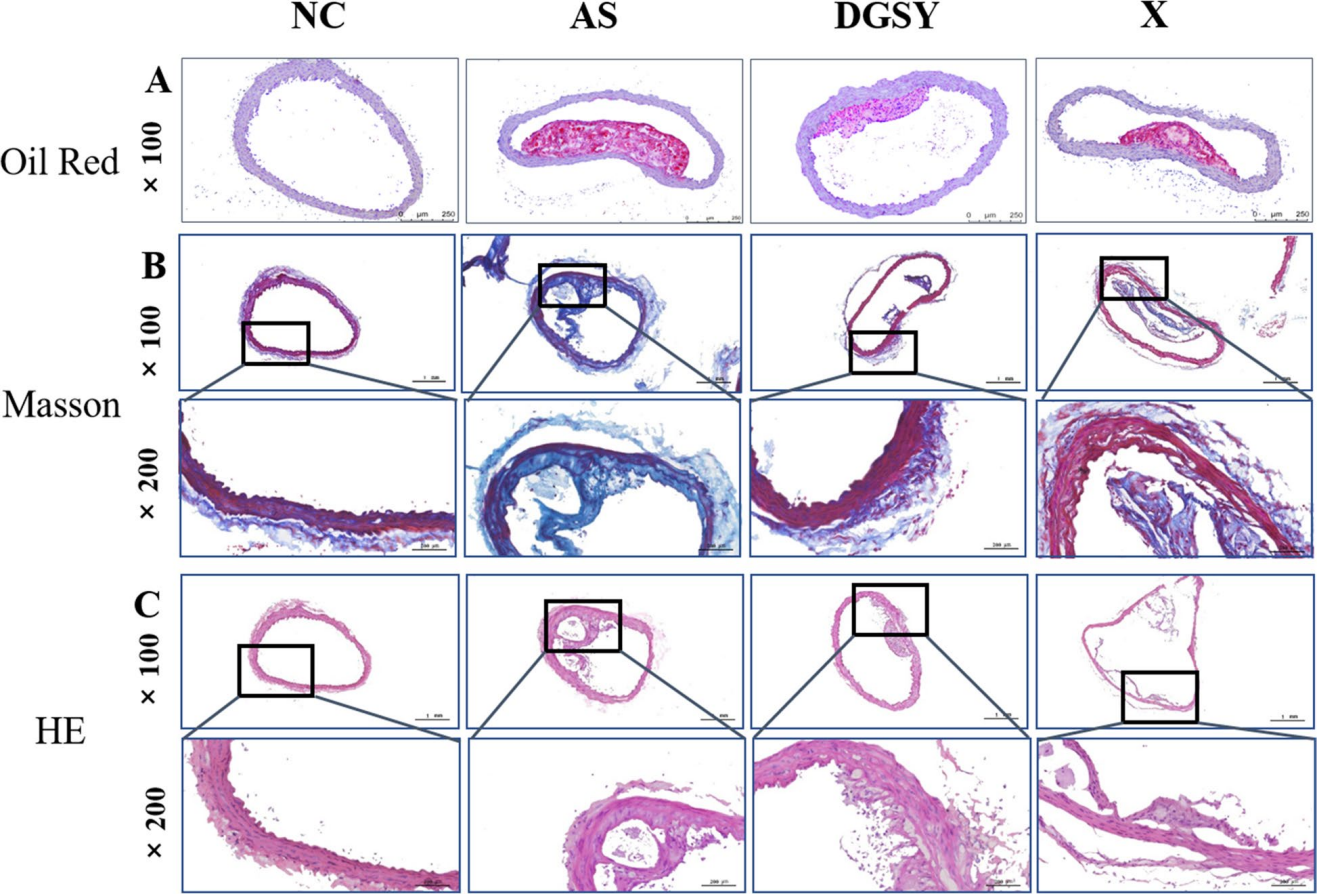
Effects of DGSY on pathological morphology of aorta in ApoE-/- mice. **A** Oil red staining (× 100), *n* = 3. **B** Masson staining (× 100, × 200), *n* = 3. **C** HE staining (× 100, × 200), *n* = 3

To observe the effect of DGSY on the morphology of aortic vessels, Oil red O, Masson, and HE stained slides were analyzed (Fig. 2). Compared with the NC group, the pathological morphology of the murine aortic vessels in the AS group had different degrees of injury. Oil red O stains showed that the aortic plaque area had significantly increased whereas Masson stains showed a decrease in aortic collagen fibers. HE staining showing significant intimal thickening of the aortic wall, disordered arrangement of endothelial cells, increased lipid deposition, and pronounced plaque protrusion into the lumen. After 16 weeks of drug intervention (Fig. 2B & C), aortic plaques were significantly reduced and aortic collagen fibers were significantly increased in DGSY and X groups. The improvement of vascular pathological morphology in the DGSY was higher compared with that of X group, indicating that DGSY effectively prevented atherosclerosis.

### Effects of DGSY on disordered lipid metabolism

**Fig. 3:**
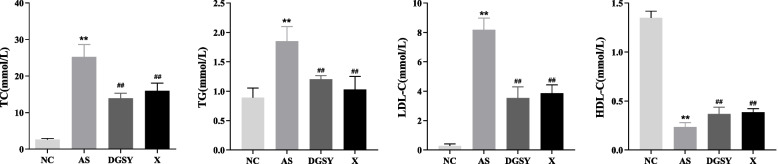
Effect of DGSY on blood lipid indexes in mice. **A** Changes in the content of TC. **B** Changes in the content of TG. **C** Changes in the content of LDL-C. **D** Changes in the content of HDL-C. The data are expressed as the means ± S.D. (*n* = 6). ^**^ *p* < 0.01 vs NC, ^##^ *p* < 0.01 vs AS

To determine the regulatory effect of DGSY on blood lipid metabolism, TC, TG, LDL-C, and HDL-C levels in the serum of mice were quantified. Levels of TC, TG, and LDL-C in serum were significantly higher (*P* < 0.01) in the M than NC group. Conversely, the levels of HDL-C were lower (*P* < 0.01) (Fig. 3). This indicated that the high-fat diet induced AS mice had typical dyslipidemia. We found that the levels of TC, TG, and LDL-C had decreased (*P* < 0.01), and HDL-C had increased (*P* < 0.01) in both DGSY and X groups. However, the difference was not significant. Thus, DGSY can improve blood lipid metabolism similar to atorvastatin calcium.

### Effects of DGSY on IL-6 and IL-8

**Fig. 4:**
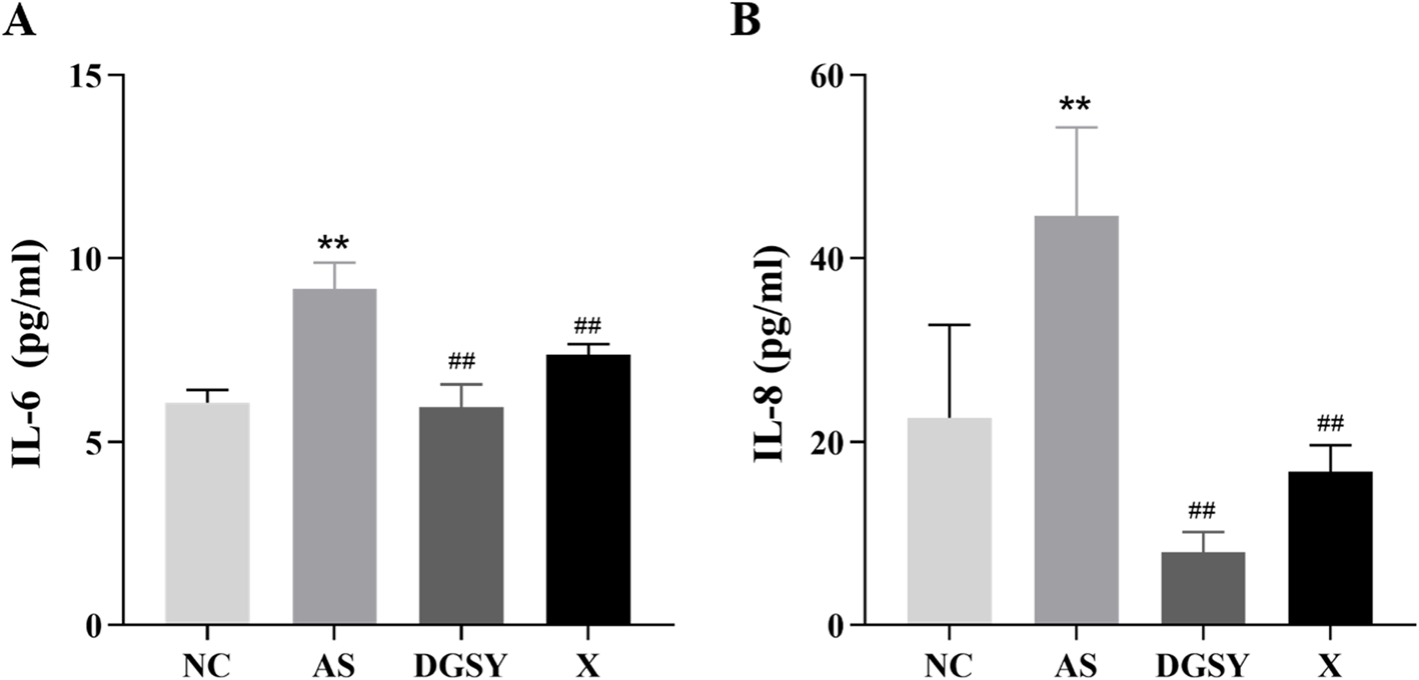
Effects of DGSY on IL-6 and IL-8 levels. **A** DGSY reduced the level of IL-6. **B** DGSY reduced the level of IL-8. The data are expressed as the means ± S.D. (*n* = 6). ^**^ *p* < 0.01 vs NC, ^##^ *p* < 0.01 vs AS

Considering that DGSY exerted anti-inflammatory effects, its impact on the levels of IL-6 and IL-8 in vascular tissues was explored with ELISA test. Results shown in Fig. 4 indicated that IL-6 and IL-8 levels were increased in the AS group, and treatment with DGSY and atorvastatin calcium reversed this effect (*P* < 0.01). Importantly, DGYS significantly reduced the levels of IL-6 and IL-8 more than atorvastatin calcium (*P* < 0.01), thus indicating that DGSY is a better relief for vascular endothelial inflammation.

### Effects of DGSY on ICAM-1 and VCAM-1

**Fig. 5:**
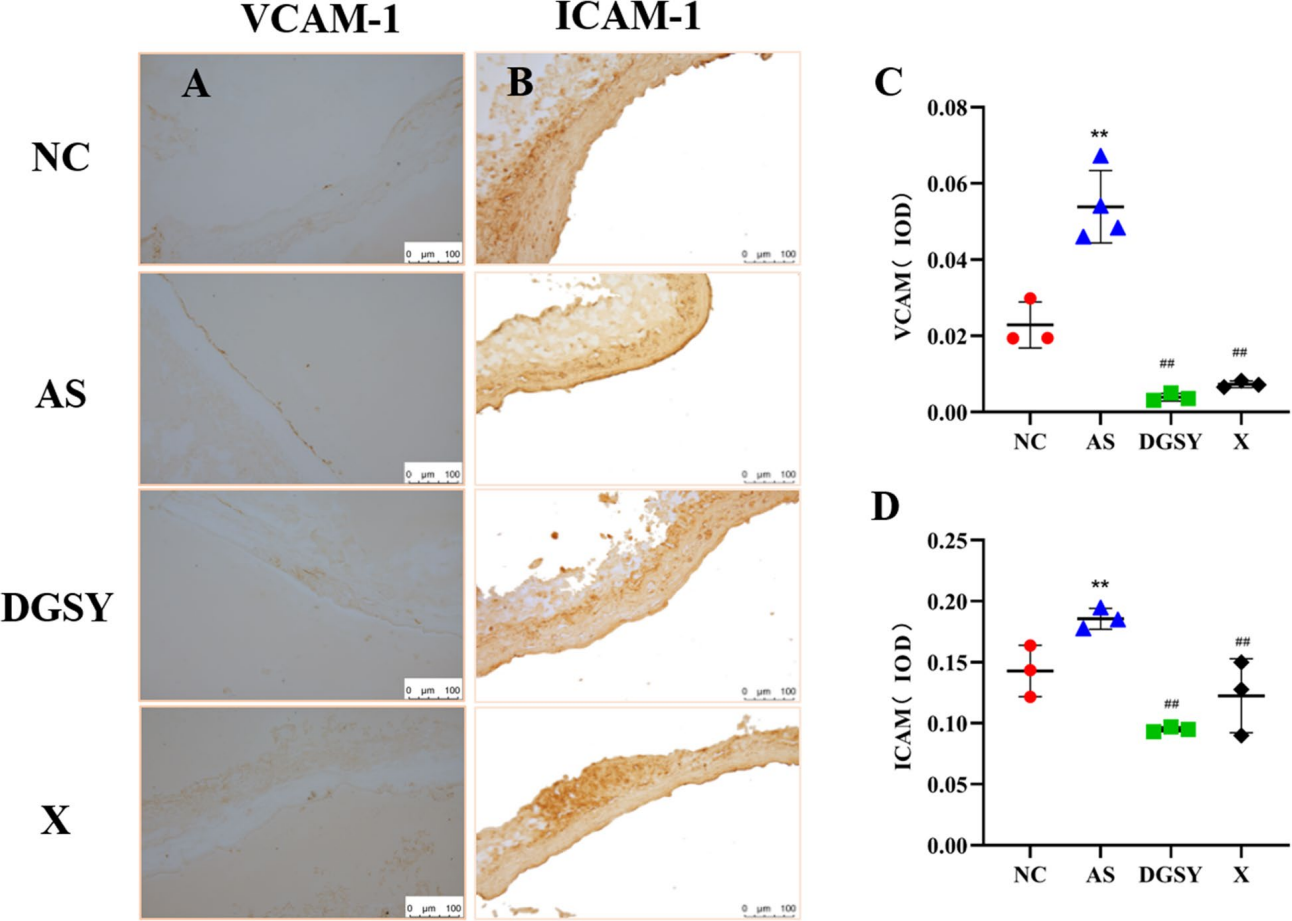
DGSY inhibited the expression of VCAM-1 and ICAM-1. **A** Expression of VCAM-1 in vascular endothelium (SP, DAB × 200), *n* = 4. **B** Expression of ICAM-1 in vascular endothelium (SP, DAB × 200), *n* = 6. **C** Bar diagram showing the expression of VCAM-1. **D** Bar diagram showing the expression of ICAM-1. The data are expressed as the means ± S.D. ^**^ *p* < 0.01 vs, ^##^ *p* < 0.01 vs AS

Activation of vascular endothelial cells is the initiating factor of AS. Endothelial cell activation triggers an inflammatory response, which mainly upregulates the expression of adhesion factors ICAM-1 and VCAM-1. Therefore, we measured the expression of VCAM-1 and ICAM-1 by immunohistochemistry (Fig. 5). Concurrently, the semi-quantitative measurement of optical density (IOD) was marked with Image-Pro Plus version 6.0 analysis software to calculate the relative expression of VCAM-1 and ICAM-1. Measure the integral absorbance of the positive expression area in the immunohistochemical sections, and calculate the average absorbance = integral absorbance/aortic cross-sectional area. The average absorbance represented the expression intensity of vascular endothelial adhesion factor in the aorta. In the NC group, the structure of aortic endothelium was clear, and there was almost no expression of VCAM-1 and ICAM-1, as determined from the yellow-stained particles. Brown-yellow particles were significantly less in the aortic endothelium in the DGSY and X groups than in the NC group (*P* < 0.01). This indicates that DGSY and atorvastatin calcium significantly decreased the expression of VCAM-1 and ICAM-1. The decrease in expression was significantly higher in the DGSY than in the X group (*P* < 0.01), indicating that the effects of DGSY were superior to those of atorvastatin calcium.

### Effects of DGSY on interα5β1, c-Abl, and YAP

**Fig. 6:**
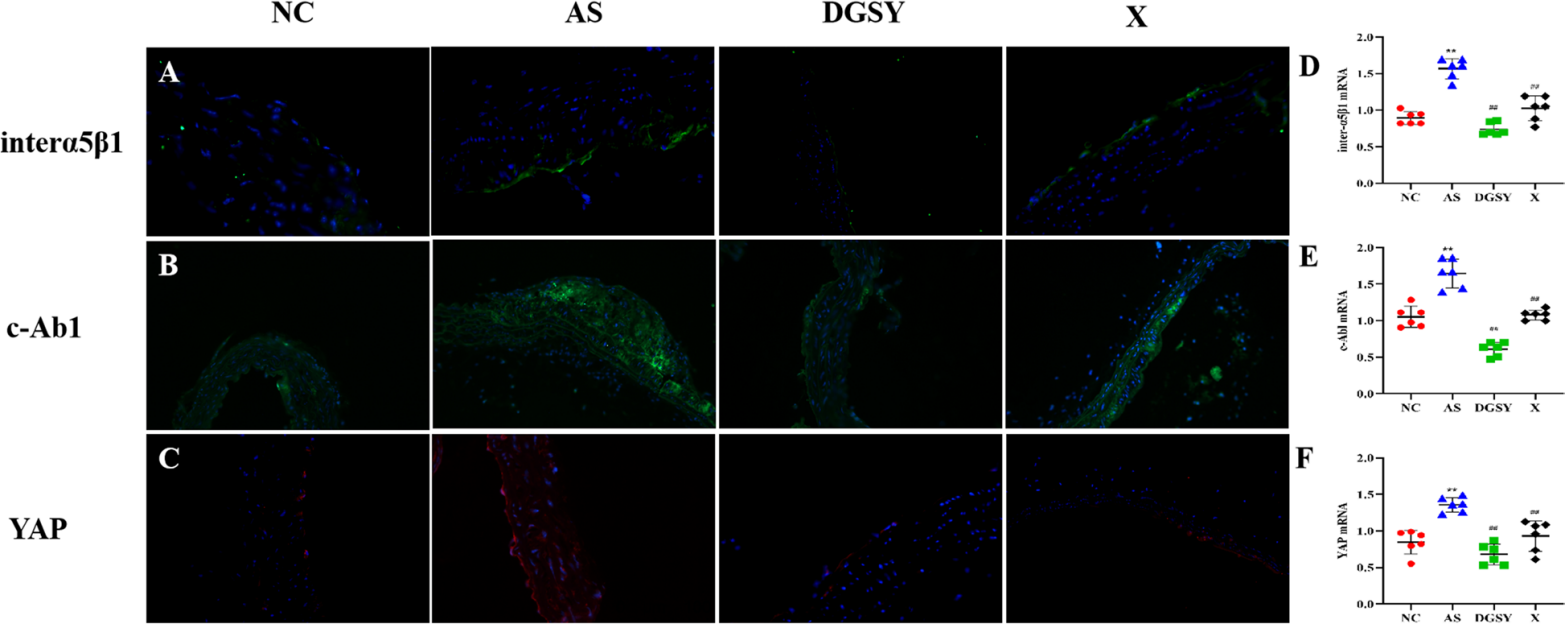
Effects of DGSY on the levels of inter-α5β1, c-Abl, and YAP in vascular endothelium. **A** Expression of interα5β1 (green) and nuclear (blue), *n* = 3. **B** Expression of c-Abl (green) and nuclear (blue), *n* = 3. **C** Expression of YAP (red) and nuclear (blue) YAP, *n* = 3. **D** The mRNA levels of interα5β1, (*n* = 6). (E) The mRNA levels of c-Abl, (*n* = 6). (F) The mRNA levels of YAP, (*n* = 6). The data are expressed as the means ± S.D. **p < 0.01 vs, ##*p* < 0.01 vs AS

To determine the effects of DGSY on interα5β1/c-Abl/YAP signal pathways (Fig. 6), their mRNA levels were measured by qT-PCR and the location of expression was assessed by immunofluorescence. The mRNA levels of *interα5β1*, *c-Ab1,* and YAP increased (*P* < 0.01) in the AS group, which was consistent with the increased expression from the immunofluorescence assays. The mRNA levels of *interα5β1*, *c-Ab1,* and *YAP* (*P* < 0.01) and expression —as assessed by immunofluorescence— were lower in the DGSY and X groups than in the NC group. Levels of interα5β1 and c-Ab1 were lower in DGSY than in the X group (*P* < 0.01). However, there was no significant difference between the two groups in YAP expression.

## Discussion

Increasing evidence has recently shown that AS is a chronic vascular inflammatory disease. The changes in the structure and function of vascular endothelial cells are the initial steps in the occurrence and development of AS. The vascular endothelium is a multifunctional endocrine organ, which can synthesize and secrete a variety of substances and regulate body processes. The early pathological changes in AS mainly depend on vascular endothelial dysfunction, and this leads to: lipid infiltration; the presence of inflammatory factors, macrophages, and smooth muscle cells accumulation in the blood vessels; damage to the vascular endothelium; loss of vascular wall elasticity; formation of yellow atheroma plaques; and upregulated expression of cell adhesion factor —these suggest that the vascular endothelium plays an important role in the pathogenesis of AS [22–24]. The damage to vascular endothelium can be reversed to some extent [25]. Therefore, protection of the vascular endothelium is an effective way to prevent and treat AS.

DGSY has a protective effect on vascular endothelial cells and plays a protective role in various pathological conditions [26, 27]. However, as with many other traditional Chinese medicines, there are few animal studies on DGSY, most of which are on either a single inflammatory cytokines or vascular adhesion cytokines. To verify the protective effect of DGSY on the vascular endothelium, the levels of blood lipids, inflammatory cytokines, and vascular cell adhesion cytokines were assessed. Moreover, our study determined the turbulence signal of inter-α5β1 /c-Abl/YAP.

Dyslipidemia is the pathological basis of AS. Increased blood lipid levels can damage vascular endothelial cells, which promotes the release of inflammatory factors that accelerate AS progression [28]. The main components of blood lipids are TC, TG, and LDL-C. Elevated blood lipid levels lead to lipid deposition and free radical release in vascular endothelial and endothelial cells respectively, which exacerbates vascular endothelial dysfunction [29]. Lipids that are predominantly LDL penetrate through vascular endothelial cells into the subcutaneous space where they undergo oxidation to OX-LDL, resulting in cell necrosis that forms atherosclerotic plaques [30]. Relatedly, Austin MA showed that TG —an independent risk factor for atherosclerotic pathogenesis— was associated with vascular endothelial injury [31]. HDL binds excess cholesterol in the body and transports it from the site of plaque damage to the liver, which protects against AS [32]. Therefore, improving lipid metabolism disorders can effectively reduce vascular endothelium damage and prevent AS. This study proved that DGSY significantly reduced TC, TG, and LDL-C (*p* < 0.01) and increased HDL-C (*p* < 0.01) levels. Interestingly, we found no significant difference in the improvement of blood lipid levels between the DGSY and X groups. Noteworthy was that this effect of DGSY was equivalent to that of atorvastatin calcium.

AS is also hypothesized as a chronic inflammation involving the arterial wall and is closely related to vascular endothelial dysfunction [33]. After arterial vascular damage, many inflammatory cytokines are released and macrophages form foam cells, which activate the NF-κB signaling pathway, which promotes the re-injury of the vascular endothelium. As a result, strips of lipids are easily formed in the vessel wall, causing the vessel to lose its elasticity and form plaques. IL-6 and IL-8 are closely related to vascular endothelial inflammation. QF Zeng demonstrated that IL-6 promoted the expression of LDL receptors in macrophages, enhancing their intake of LDL, thus promoting the formation of foam cells and upregulated expression of adhesion factors and other cytokines [34]. Azghania showed that IL-8 promoted: the accumulation of neutrophils; the tight adhesion of monocytes to endothelial cells; and the invasion of the vascular wall. These aggravated vascular endothelial cell damage [35]. In contrast, macrophages, and damaged endothelial cells release interleukin factors such as IL-6 and IL-8, which exacerbate the inflammatory cascade. Therefore, we evaluated the infiltration of vascular endothelial inflammation by IL-6 and IL-8. DGSY reduced IL-6 and IL-8 levels, suggesting it effectively controlled both the development of inflammation and reduced the inflammatory infiltration of vascular endothelium. Interestingly, DGSY was better in this respect than atorvastatin calcium.

Hemodynamics can affect the function of vascular endothelial cells. Laminar flow and atherosclerotic plaques preferentially develop at branches and curvatures in the arterial system where flow is either turbulent or at low speed [36]. At the cellular and molecular levels, increased abnormal shear stress regulated endothelial inflammation, including the expression of VCAM-1 and ICAM-1. This activated MAPKs, NF-κB, YAP/YAZ, and other signaling pathways to damage vascular endothelial cells [37–40]. VCAM-1 and ICAM-1 promoted the adhesion and migration of inflammatory cells to endothelial cells and the transformation of monocytes to macrophages and foam cells [41]. Conversely, inhibition of VCAM-1 and ICAM-1 reduced endothelial cell activity and attenuated endothelial injury and inflammation [42]. Our study showed that DGSY downregulated the expression of VCAM-1 and ICAM-1, which reduced vascular endothelium damage corroborating previous studies [16]. Moreover, the expression of VCAM-1 and ICAM-1 showed lower for DGSY group than the X group, but there was no great difference in the two groups.

Studies have shown that the interα5β1/c-Abl/YAP signaling pathway is a potential therapeutic target for the treatment of early atherosclerosis [43]. Interα5 and YAP are mechanical force receptors on endothelial cells —interα5 is especially sensitive to shear stress [44]. Turbulence can significantly increase the expression of interα5 in the activated state, triggering proinflammatory responses and proatherogenic phenotypes in vascular endothelial cells. Relatedly, endothelial-specific overexpression of YAP increased the degree of atherosclerosis in mice [45]. As a key upstream factor of turbulence regulation, interα5 can induce YAP to accumulate continuously in the nucleus, upregulate the expression of ICAM-1 and VCAM-1 and promote the proliferation and inflammatory phenotype of endothelial cells.

The c-Abl can activate interα5β1/YAP, and interα5 activation increases c-Abl activity, resulting in phosphorylation of the YAP Y357 residue and subsequent increase in YAP stability [46]. Studies have shown that the inhibitor of c-Abl significantly reduced the phosphorylation of YAP tyrosine 357 following activation by interα5β1. This in turn decreases endothelial inflammation and plaque formation. In our study, the expression of interα5β1,c-Abl, and YAP were used to indicate vascular endothelium damage. Levels of interα5β1/YAP mediated by c-Abl decreased in the vascular tissues of mice in the DGSY group, which was consistent with the immunofluorescence assays, indicating that DGSY significantly alleviated vascular endothelial injury. The reduction of interα5β1,c-Abl, and YAP levels were more obvious in DGSY than in atorvastatin calcium treatment groups, suggesting that DGSY is beneficial for reducing vascular injuries caused by blood flow. Moreover, DGSY improved the function of vascular cells by inhibiting MAPKs and IKK/NF-κB signaling pathways, reducing levels of TNF-α, IL-6, IL-1β, and Cox-2 [47].

## Conclusions

In conclusion, our study has demonstrated the potential of DGSY in protecting the vascular endothelium and alleviating AS by modulating expression levels of TC, TG, LDL-C, and HDL-C, reducing IL-6 and IL-8 expression level, inhibiting ICAM-1 and VCAM-1 expression, and suppressing the expression of interα5β1 /c-Abl/YAP signaling pathway. It's worth noting that the effect of DGSY was better than that of atorvastatin calcium which need enlarge the sample size to confirm it in the next step.

## Data Availability

The datasets presented in the current study are available from the corresponding author upon reasonable request.
